# Infection detection revolution: Harnessing AI-powered image analysis to combat infectious diseases

**DOI:** 10.1371/journal.pone.0307437

**Published:** 2024-10-09

**Authors:** Muhammad Ahsan, Robertas Damaševičius

**Affiliations:** Faculty of Informatics, Vytautas Magnus University, Kaunas, Lithuania; State University of New York at Oswego, UNITED STATES OF AMERICA

## Abstract

Infectious diseases wield significant influence on global mortality rates, largely due to the challenge of gauging their severity owing to diverse symptomatology. Each nation grapples with its unique obstacles in combatting these diseases. This study delves into three distinct decision-making methodologies for medical diagnostics employing Neutrosophic Hypersoft Set (NHSS) and Plithogenic Hypersoft Set (PHSS), extensions of the Hypersoft set. It introduces state-of-the-art AI-driven techniques to enhance the precision of medical diagnostics through the analysis of medical imagery. By transforming these images into the aforementioned sets, the analysis becomes more refined, facilitating more accurate diagnoses. The study advocates various courses of action, including isolation, home or specialized center quarantine, or hospitalization for further treatment. The novelty in this study utilizes cutting-edge AI methods to enhance medical imaging, transforming them into accurate diagnostic tools, marking a significant change in how infectious diseases are addressed. By combining machine learning and pattern recognition, it offers the potential to overhaul healthcare worldwide, facilitating accurate diagnoses and customized treatment plans, ultimately reducing the global burden of infectious diseases on mortality rates.

## 1 Introduction

Infectious diseases are caused by pathogenic microorganisms such as viruses, bacteria, fungi, and parasites, as described in references [1, 2]. These pathogens can spread from one individual to another, either directly or indirectly, posing a significant global public health threat. Annually, these diseases result in the deaths of millions across various ages, genders, and socioeconomic statuses. The severity of these conditions varies, ranging from mild to critical, with some diseases potentially being fatal if not appropriately managed. Modes of transmission include inhalation of respiratory droplets, ingestion of contaminated food or water, insect bites, or physical contact. Adopting preventive measures like hand washing, vaccinations, and adherence to infection control protocols can reduce the risk of spread. While vaccines and specific treatments such as antibiotics and antivirals can prevent or cure many infectious diseases, the emergence of drug-resistant strains and the absence of effective treatments for other conditions underscore the urgent need for continued research in disease prevention, diagnosis, and treatment. Such research is crucial for reducing the substantial burden of infectious diseases on a global scale. In healthcare, making informed decisions is critical, especially in diagnosing and treating patients, as noted in references [3, 4]. Healthcare professionals must base their decisions on a thorough evaluation of a patient’s medical history, observed symptoms, and diagnostic tests to optimize care and enhance recovery prospects. Effective decision-making is essential for early diagnosis, minimizing complications, and ensuring timely and appropriate treatment. This process is intricate, considering patient preferences, clinical guidelines, and resource availability, and it involves assessing the benefits and risks of treatment options based on solid evidence. The integration of machine learning and AI technologies [5, 6] is becoming increasingly significant in healthcare decision-making, allowing for the analysis of extensive data sets to tailor treatments to individual patient needs. Effective medical decision-making requires a collaborative approach among healthcare providers, patients, and their families to reach informed, consensus-based decisions. Open communication about treatment options and their potential outcomes is crucial. Ongoing research into decision-making methodologies, models, and frameworks is vital for advancing healthcare quality and patient outcomes. Soft sets, introduced by Molodtsov in 1999, are tools that address uncertainty in a parameterized manner [7]. However, they encounter limitations when parameters exhibit sub-parametric types of uncertain and unclear data values. To address this issue, Musa introduced the concept of Bipolar hypersoft sets in 2021, which extends soft sets to better handle uncertain or incomplete information in two dimensions by incorporating these intricate types of data [8]. In the field of data mining, Hypersoft Set (HSS) is crucial for classifying and analyzing data characterized by uncertain or incomplete attributes, thus facilitating the discovery of novel and useful patterns. Within expert systems, HSS aids in the representation and analysis of expert knowledge, which contributes to the creation of more precise and effective systems. In medical diagnostics, the implementation of HSS has enhanced the accuracy of diagnoses [9]. Additionally, in image processing, HSS significantly improves the management of uncertain or imprecise information by considering the sub-parametric nature of data, which in turn boosts the accuracy and efficiency of image segmentation and classification algorithms. This study delves into three distinct decision-making methodologies for medical diagnostics employing Neutrosophic Hypersoft Set (NHSS) and Plithogenic Hypersoft Set (PHSS), extensions of the Hypersoft set. It introduces state-of-the-art AI-driven techniques to enhance the precision of medical diagnostics through the analysis of medical imagery. By transforming these images into the aforementioned sets, the analysis becomes more refined, facilitating more accurate diagnoses. The study advocates various courses of action, including isolation, home or specialized center quarantine, or hospitalization for further treatment. The novelty in this study utilizes cutting-edge AI methods to enhance medical imaging, transforming them into accurate diagnostic tools, marking a significant change in how infectious diseases are addressed. By combining machine learning and pattern recognition, it offers the potential to overhaul healthcare worldwide, facilitating accurate diagnoses and customized treatment plans, ultimately reducing the global burden of infectious diseases on mortality rates.

## 2 Related works

In the field of medical image processing, enhancing the quality and information content of images by combining different imaging techniques is a key area of research. The existing literature showcases a variety of methods, from basic averaging to advanced extreme value selection. While averaging tends to reduce image contrast, extreme value methods offer only minimal enhancements. The Brovey technique is noted for causing color distortions in merged images [10]. Similarly, using intensity-hue saturation (IHS) combined with principal component analysis (PCA) leads to lower image quality and spatial distortions [11]. Although pyramid decomposition enhances spectral details, it fails to adequately preserve edges [12]. Methods like the discrete cosine transform (DCT) and singular value decomposition (SVD) create more comprehensive images but struggle with accurately delineating tumor boundaries [13, 14]. The discrete wavelet transform (DWT) improves time-frequency localization but suffers from shift-variance due to downsampling [15], a drawback somewhat mitigated by the more sophisticated redundant wavelet transform (RWT), which still falls short in edge detailing [16]. The contourlet transform offers better edge detailing but lacks shift invariance, essential for applications like watermarking and image fusion [17–21]. This problem is resolved by the non-subsampled contourlet transform (NSCT) and the non-subsampled Shearlet transform (NSST), which eliminate such deficiencies [22–24]. However, the combination of DWT and fuzzy logic can result in reduced contrast due to uncertainties in the fusion process [25]. Challenges in medical imaging such as inadequate lighting often lead to poor contrast and reduced visibility, complicating disease diagnosis due to increased uncertainties and ambiguities. Although techniques like gray-level adjustments and histogram equalization have been explored [26, 27], they do not fully rectify image quality issues. The introduction of fuzzy sets by Zadeh in 1965 began to address these ambiguities, though not without leaving some uncertainties [28]. This was further developed by Atanassov in 1986 through the creation of the Intuitionistic Fuzzy Set (IFS), which introduces a third element hesitation to the concepts of membership and non-membership, providing a more comprehensive handling of uncertainties [29]. Recent advancements include a method that quantifies pixel uncertainty using a novel fuzzy metric and integrates this within a fuzzy hierarchical fusion attention neural network that employs multiscale guided learning. This approach transforms images into the fuzzy domain, utilizing custom fuzzy rules and neural network convolutions [30]. Additionally, the Fuzzy DBNet, which combines the dual butterfly network with a fuzzy atrous spatial pyramid pooling in a deep learning framework, shows remarkable segmentation accuracy in datasets including pills and lung X-rays [31]. Another significant advancement is a multi-objective medical image fusion model that merges low-frequency components via averaging and high-frequency components using an optimized Type-2 fuzzy entropy method, further refined by the Adaptive Electric Fish Optimization (A-EFO) algorithm [32]. These innovations signify substantial progress in medical imaging enhancement techniques.

## 3 Methodologies

In this section, three methodologies are introduced, utilizing hypothetical (synthetic) data as a prototype. The experts recruited for this study are mathematicians, and these methodologies can be applied to real data in the future.

**Definition 1** [8] Let A and B be Neutrosophic Hypersoft Sets (NHSS) defined over the universe of discourse $X=\{\zeta_{1},\zeta_{2},\zeta_{3},\ldots ,\zeta_{n}\}$, with m distinct attributes denoted by $\varepsilon_{1},\varepsilon_{2},\varepsilon_{3},\ldots ,\varepsilon_{m}$, where m≥1. The characteristic values corresponding to these attributes are represented by the sets ${ð}_{1},{ð}_{2},{ð}_{3},\ldots ,{ð}_{m}$. We introduce a function $H:{ð}_{1}\times {ð}_{2}\times {ð}_{3}\times \ldots \times {ð}_{m}\to ℘\left(X\right)$, where ℘(X) denotes the power set of X. The NHSS approximate functions for A and B are given by:

$$\begin{array}{c} \gamma_{A}\left(\varepsilon_{i}\right)=\{\left(\zeta ,\omega_{A}\left(\zeta \right),\sigma_{A}\left(\zeta \right),\beta_{A}\left(\zeta \right)\right):\zeta \in X\} \end{array}$$

$$\begin{array}{c} \lambda_{B}\left(\varepsilon_{i}\right)=\{\left(\zeta ,\omega_{B}\left(\zeta \right),\sigma_{B}\left(\zeta \right),\beta_{B}\left(\zeta \right)\right):\zeta \in X\} \end{array}$$

Here, $\omega_{A}\left(\zeta \right)$, $\sigma_{A}\left(\zeta \right)$, $\beta_{A}\left(\zeta \right)$, $\omega_{B}\left(\zeta \right)$, $\sigma_{B}\left(\zeta \right)$, and $\beta_{B}\left(\zeta \right)$ represent the membership, indeterminacy, and non-membership grades of *ζ* in A and B respectively, for attribute *ϵ*_*i*_. The distance between A and B is determined as follows:

1. For Hamming Distance (HD), the distance is defined by: $d_{NHSS}^{s}\left(A,B\right)$
  $$\begin{array}{c} =\frac{1}{2m}\{\sum_{i=1}^{m}\sum_{j=1}^{n}|\omega_{A}\left(\zeta_{j}\right)-\omega_{B}\left(\zeta_{j}\right)|+|\sigma_{A}\left(\zeta_{j}\right)-\sigma_{B}\left(\zeta_{j}\right)|+|\beta_{A}\left(\zeta_{j}\right)-\beta_{B}\left(\zeta_{j}\right)|\} \end{array}$$ (1)
  Where *ζ*_*j*_ denotes the *j*-th element of X.

**Example 1** Let $X=\{\zeta_{1},\zeta_{2},\zeta_{3}\}$ be the universe of discourse with attributes *ϵ*_1_ = Color and *ϵ*_2_ = Size, and characteristic values ${ð}_{1}=\left\{\text{Red},\text{Blue}\right\}$, ${ð}_{2}=\left\{\text{Small},\text{Large}\right\}$. Define NHSS for A and B:

$$\begin{array}{c} \gamma_{A}\left(\epsilon_{1}\right)=\{(\zeta ,0.7,0.2,0.1):\zeta \in X\} \end{array}$$

$$\begin{array}{c} \lambda_{B}\left(\epsilon_{1}\right)=\{(\zeta ,0.5,0.4,0.1):\zeta \in X\} \end{array}$$

The Hamming distance between A and B is:

$$\begin{array}{cc} d_{\text{NHSS}}^{s}\left(A,B\right) & =\frac{1}{2}\sum_{j=1}^{3}\left(|0.7-0.5|+|0.2-0.4|+|0.1-0.1\right|\\ & \;+|0.6-0.7|+|0.3-0.1|+|0.1-0.2|+|0.4-0.6|\\ & \;+|0.3-0.2|+|0.3-0.2|)=0.6 \end{array}$$ (2)

**Definition 2** [33] Given the context of NHSS defined over a universal set U, let ${ϒ}_{A}$ and $\Psi_{B}$ symbolize two such spaces. The Similarity Measure (SM) between these spaces, facilitated by the application of the Hamming Distance (HD), introduces an elegant formulation. Consider the expressions below, which articulate the SM in two distinct manners:The first similarity measure, employing the inverse of the sum with unity and the scaled HD, is given by:

$$\begin{array}{c} S_{NHSS}^{\prime }\left(ϒA,\Psi_{B}\right)=\frac{1}{1+d_{NHSS}^{s}\left({ϒ}_{A},\Psi_{B}\right)} \end{array}$$ (3)

**Definition 3** [34] The newly introduced Plithogenic Distance Measures (PDM) include the Plithogenic Hamming Distance Measure ($d_{H}^{R}$), the Normalized Plithogenic Hamming Distance Measure ($d_{NH}^{R}$), the Plithogenic Euclidean Distance Measure ($d_{E}^{R}$), and the Normalized Plithogenic Euclidean Distance Measure ($d_{NE}^{R}$). These measures are defined for calculating the distance between two Plithogenic Hypersoft Sets (PHSSs), denoted as R1 and R2. The mathematical formulations for these distances are provided below: For the Plithogenic Hamming Distance (PHD) Measure:

$$\begin{array}{c} d_{H}^{R}(R1,R2)=\frac{1}{m}\sum_{i=1}^{m}\sum_{j=1}^{l}|d_{R1}^{i}(\delta j)-d_{R2}^{i}(\delta j)|\cdot \text{max}(c_{F}^{i}(\delta_{j},\delta_{d})) \end{array}$$ (4)

For the Normalized Plithogenic Hamming Distance (NPHD) Measure:

$$\begin{array}{c} d_{NH}^{R}(R1,R2)=\frac{1}{mn}\sum_{i=1}^{m}\sum_{j=1}^{l}|d_{R1}^{i}(\delta j)-d_{R2}^{i}(\delta j)|\cdot \text{max}(c_{F}^{i}(\delta_{j},\delta_{d})) \end{array}$$ (5)

For the Plithogenic Euclidean Distance (PED) Measure:

$$\begin{array}{c} d_{E}^{R}(R1,R2)={\left[\frac{1}{m}\sum_{i=1}^{m}\sum_{j=1}^{l}{\left(d_{R1}^{i}(\delta j)-d_{R2}^{i}(\delta j)\right)}^{2}\cdot \text{max}(c_{F}^{i}(\delta_{j},\delta_{d}))\right]}^{\frac{1}{2}} \end{array}$$ (6)

For the Normalized Plithogenic Euclidean Distance (NPED) Measure:

$$\begin{array}{c} d_{NE}^{R}(R1,R2)=\frac{1}{n}{\left[\frac{1}{m}\sum_{i=1}^{m}\sum_{j=1}^{l}{\left(d_{R1}^{i}(\delta j)-d_{R2}^{i}(\delta j)\right)}^{2}\cdot \text{max}(c_{F}^{i}(\delta_{j},\delta_{d}))\right]}^{\frac{1}{2}} \end{array}$$ (7)

These formulations serve as the foundation for quantifying the dissimilarity between any two given PHSSs, employing various methods to accommodate the characteristics of the data under analysis.

**Definition 4** [34] A newly proposed concept, termed Pythagorean Similarity Measure (PSM), is predicated on the innovative development of Pythagorean Distance Measure (PDM). This relationship is mathematically articulated as:

$$\begin{array}{c} M^{R}\left(R_{1},R_{2}\right)=\frac{1}{1+d^{R}\left(R_{1},R_{2}\right)} \end{array}$$

In this context, $d^{R}\left(R_{1},R_{2}\right)$ signifies the plithogenic distance. Notably, this distance can be classified into several types, including: the plithogenic Hamming distance $d_{H}^{R}\left(R_{1},R_{2}\right)$, the normalized plithogenic Hamming distance $d_{NH}^{R}\left(R_{1},R_{2}\right)$, the plithogenic Euclidean distance $d_{E}^{R}\left(R_{1},R_{2}\right)$, or the normalized plithogenic Euclidean distance $d_{NE}^{R}\left(R_{1},R_{2}\right)$.

### 3.1 Methodology I

#### 3.1.1 Application I

In this particular application, our primary aim is the diagnosis of SARS in specific patients by determining their infection status. To accomplish this, we initially acquire chest images of the patient. We acquire chest X-ray images of individuals under suspicious and confirm SARS case from the Kaggle (https://www.kaggle.com/datasets/yazanqiblawey/sars-mers-xray-images-dataset) platform and apply the methodology detailed in Step 2 to transform these images into NHSS format. For the purpose of comparative analysis, an NHSS representative of a confirmed SARS case is pre-stored within the system. By employing a similarity assessment technique, we scrutinize the correlation between the patient’s NHSS and the SARS reference NHSS archived in our database. A significant resemblance between the two NHSS profiles indicates a probable SARS infection, thereby classifying the individual as a potential SARS case. To facilitate rapid diagnostics and enable local treatment of patients in a cost-effective manner, we propose the creation of software designed as an online tool. This tool would cater to a domain of discussion involving two individuals under suspicion, denoted as $X=\{\zeta_{1},\zeta_{2}\}$. Here, the set of parameters ${ð}_{1},{ð}_{2}$, and ${ð}_{3}$ represent certain perceptible symptoms, specifically: ${ð}_{1}=\left\{\varkappa_{1},\varkappa_{2}\right\}$ (Fever, Cough), ${ð}_{2}=\left\{\varkappa_{3},\varkappa_{4}\right\}$ (Fatigue, Sore throat), and ${ð}_{3}=\left\{\varkappa_{5},\varkappa_{6}\right\}$ (Vomiting, Headache). Additionally, we define the Cartesian product of these sets as $G={ð}_{1}\times {ð}_{2}\times {ð}_{3}$, yielding $G=\{g_{1},g_{2},g3,\ldots ,g8\}$ where each $g_{i},i\in 1,2,3,\ldots ,8$ represents a 3-tuplet encapsulating various combinations of symptoms.

**Stage 1:** Following this, we construct two NHSS frameworks with the guidance of healthcare experts, specifically designed for individuals suspected of having SARS, as described below:
  $$\begin{array}{c} \Gamma_{A}=\{\begin{array}{cc} \{ & (g_{1},\left\{\left(\zeta_{1},0.1,0.6,0.3\right),\left(\zeta_{2},0.7,0.2,0.2\right)\right\}),\\ & (g_{2},\left\{\left(\zeta_{1},0.4,0.1,0.2\right),\left(\zeta_{2},0.6,0.2,0.4\right)\right\}),\\ & (g_{3},\left\{\left(\zeta_{1},0.3,0.3,0.1\right),\left(\zeta_{2},0.2,0.7,0.1\right)\right\}),\\ & (g_{4},\left\{\left(\zeta_{1},0.1,0.1,0.2\right),\left(\zeta_{2},0.1,0.2,0.5\right)\right\}),\\ & (g_{5},\left\{\left(\zeta_{1},0.6,0.4,0.2\right),\left(\zeta_{2},0.4,0.5,0.6\right)\right\}),\\ & (g_{6},\left\{\left(\zeta_{1},0.2,0.6,0.6\right),\left(\zeta_{2},0.1,0.5,0.3\right)\right\}),\\ & (g_{7},\left\{\left(\zeta_{1},0.4,0.2,0.8\right),\left(\zeta_{2},0.4,0.2,0.4\right)\right\}),\\ & (g_{8},\left\{\left(\zeta_{1},0.1,0.7,0.5\right),\left(\zeta_{2},0.3,0.2,0.4\right)\right\})\}. \end{array}\}. \end{array}$$ (8)
  and
  $$\begin{array}{c} \Lambda_{B}=\{\begin{array}{cc} \{ & (g_{1},\left\{\left(\zeta_{1},0.6,0.1,0.5\right),\left(\zeta_{2},0.4,0.1,0.5\right)\right\}),\\ & (g_{2},\left\{\left(\zeta_{1},0.7,0.1,0.4\right),\left(\zeta_{2},0.2,0.7,0.2\right)\right\}),\\ & (g_{3},\left\{\left(\zeta_{1},0.6,0.1,0.5\right),\left(\zeta_{2},0.1,0.4,0.7\right)\right\}),\\ & (g_{4},\left\{\left(\zeta_{1},0.1,0.5,0.4\right),\left(\zeta_{2},0.2,0.3,0.2\right)\right\}),\\ & (g_{5},\left\{\left(\zeta_{1},0.2,0.7,0.5\right),\left(\zeta_{2},0.8,0.4,0.5\right)\right\}),\\ & (g_{6},\left\{\left(\zeta_{1},0.7,0.2,0.5\right),\left(\zeta_{2},0.2,0.7,0.5\right)\right\}),\\ & (g_{7},\left\{\left(\zeta_{1},0.6,0.1,0.8\right),\left(\zeta_{2},0.2,0.9,0.5\right)\right\}),\\ & (g_{8},\left\{\left(\zeta_{1},0.2,0.3,0.5\right),\left(\zeta_{2},0.2,0.1,0.8\right)\right\})\}. \end{array}\}. \end{array}$$ (9)
**Stage 2:** The system is described as follows, with another NHSS already included in the system as a reference point:
  $$\begin{array}{c} {ϒ}_{C}=\{\begin{array}{cc} & (g_{1},\left\{\left(\zeta_{1},0.1,0.5,0.2\right),\left(\zeta_{2},0.4,0.2,0.7\right)\right\}),\\ & (g_{2},\left\{\left(\zeta_{1},0.1,0.3,0.4\right),\left(\zeta_{2},0.4,0.3,0.7\right)\right\}),\\ & (g_{3},\left\{\left(\zeta_{1},0.4,0.5,0.6\right),\left(\zeta_{2},0.2,0.4,0.5\right)\right\}),\\ & (g_{4},\left\{\left(\zeta_{1},0.1,0.4,0.6\right),\left(\zeta_{2},0.5,0.3,0.8\right)\right\}),\\ & (g_{5},\left\{\left(\zeta_{1},0.2,0.1,0.4\right),\left(\zeta_{2},0.5,0.4,0.6\right)\right\}),\\ & (g_{6},\left\{\left(\zeta_{1},0.4,0.5,0.4\right),\left(\zeta_{2},0.2,0.4,0.5\right)\right\}),\\ & (g_{7},\left\{\left(\zeta_{1},0.5,0.3,0.6\right),\left(\zeta_{2},0.4,0.4,0.6\right)\right\}),\\ & \left(g_{8},\left\{\left(\zeta_{1},0.7,0.2,0.5\right),\left(\zeta_{2},0.3,0.5,0.5\right)\right\}\right) \end{array}\}. \end{array}$$ (10)
**Stage 3-4:** **(i)** Calculate the Hamming distances followed by determining the similarity measure among them: $\Gamma_{A}$ and ${ϒ}_{C}$:
  $$\begin{array}{c} S_{\text{NHSS}}^{\prime }\left(\Gamma_{A},{ϒ}_{C}\right)=\frac{1}{1+d_{\text{NHSS}}^{s}\left(\Gamma_{A},{ϒ}_{C}\right)}\approx 0.68>\frac{1}{2} \end{array}$$
  **(ii)** Computing the similarity measure between $\Lambda_{B}$ and ${ϒ}_{C}$:
  $$\begin{array}{c} S_{\text{NHSS}}^{\prime }\left(\Lambda_{B},{ϒ}_{C}\right)=\frac{1}{1+d_{\text{NHSS}}^{s}\left(\Lambda_{B},{ϒ}_{C}\right)}\approx 0.67>\frac{1}{2} \end{array}$$
**Stage 5:** Therefore, both sets of symptoms $\Gamma_{A}$ and ${ϒ}_{C}$, as well as $\Lambda_{B}$ and ${ϒ}_{C}$, exhibit significant similarity. Consequently, it can be inferred that the individual may potentially be afflicted with SARS.

**Algorithm 1** Diagnostic Algorithm

1: **Input:** Registered input images *I*

2: **Output:** Predicted diagnostic results

3: Read the registered input images *I*

4: **Step 1: Fuzzification**

5: The initial input image undergoes fuzzification through the application of Eq 22.

$$\begin{array}{c} \mu_{I_{1}}\left(I_{gh_{1}}\right)=\frac{I_{gh_{1}}-I_{min}}{I_{max}-I_{min}}, \end{array}$$ (11)

where $I_{gh_{1}}$ is the gray pixel of the first input image, *I*_*max*_ and *I*_*min*_ represent the highest and least gray level pixel values of the first input image, respectively.

6: **Step 2: Develop Neutrosophic Hypersoft Set (NHSS)**

7: To develop an NHSS based on the provided images:

- **Membership, Non-membership, and Indeterminacy Degree Construction:** The degree construction is defined by:
  $$\begin{array}{c} \left(\mu_{I_{1}}^{NIFS},\nu_{I_{1}}^{NIFS},\gamma_{I_{1}}^{NIFS}\right):\left(I_{gh_{1}};\beta_{opt1}\right)\mapsto \left[0,1\right], \end{array}$$ (12)
  $$\begin{array}{c} 0\leq \mu_{I_{1}}^{NIFS}+\nu_{I_{1}}^{NIFS}+\gamma_{I_{1}}^{NIFS}\leq 3 \end{array}$$ (13)
  $$\begin{array}{c} \left(I_{gh_{1}};\beta_{opt1}\right)=\frac{\left(1+\beta_{opt1}\left(1+e^{-\beta_{opt1}}\right)\right)\mu_{I_{1}}\left(I_{gh1}\right)}{\left(1+\beta_{opt1}\left(1+e^{-\beta_{opt1}}\right)\right)\mu_{I_{1}}\left(I_{gh1}\right)} \end{array}$$ (14)
- **System Representation:** The system can be represented as:
  $$\begin{array}{c} I_{IF_{1}}=\left\{I_{gh_{1}},\mu_{I_{1}}^{NIFS}\left(I_{gh_{1}};\beta_{opt1}\right),\nu_{I_{1}}^{NIFS}\left(I_{gh_{1}};\beta_{opt1}\right),\gamma_{I_{1}}^{NIFS}\left(I_{gh_{1}};\beta_{opt1}\right)\right\} \end{array}$$ (15)
- **Optimization Parameter:** The optimal parameter *β*_*opt*1_ is determined by maximizing the Pythagorean Fuzzy Entropy (PFE):
  $$\begin{array}{c} \beta_{opt1}=\underset{\beta }{\text{max}}\left(PFE\left(F;\beta \right)\right) \end{array}$$ (16)
  where PFE is calculated as:
  $$\begin{array}{c} PFE\left(F;\beta \right)=\frac{1}{n}\sum_{i=1}^{n}\pi_{F}\left(p_{i}\right)e^{\pi_{F}\left(p_{i}\right)} \end{array}$$ (17)

8: **Step 3: Calculate Distance Measure**

9: Calculate the distance measure between two sets.

10: **Step 4: Calculate Similarity Measure**

11: Calculate the similarity measure.

12: **Step 5: Predict Results**

13: Use the similarity measure to predict results.

### 3.2 Methodology II

#### 3.2.1 Application II

In the scope of our research, we have identified three subjects, labeled as $X=\{\zeta_{1},\zeta_{2},\zeta_{3}\}$, for examination regarding possible Hepatitis infection. We procure chest X-ray images from individuals suspected of having Hepatitis from the Kaggle platform here and use the method described in Step 2 to convert these images into NHSS format. For additional information. Our strategy is founded on diagnosing the presence of infection in these subjects by generating patient-specific NHSS models, $I\zeta_{i}$, designed according to each subject’s unique health profile. We plan to compare these models with a standard optimal Hepatitis NHSS model, Iideal, stored in our database, which epitomizes the immune response signature typical of Hepatitis infection. To evaluate how closely each subject’s NHSS model matches the ideal Hepatitis NHSS model, $I_{\text{ideal}}$, we will use a similarity metric. A similarity score above 0.5 will suggest a probable Hepatitis infection, prompting the need for additional medical examination. Our analytical framework is further enriched by the delineation of symptomatic parameters across four distinct sets: $S_{1},S_{2},S_{3}$, and $S_{4}$. These sets encapsulate a range of observable symptoms, with $S_{1}=\left\{s_{1},s_{2}\right\}$, $S_{2}=\left\{s_{3},s_{4}\right\}$, $S_{3}=\left\{s_{5}\right\}$, and $S_{4}=\left\{s_{6},s_{7},s_{8}\right\}$. The symptoms are respectively identified as: $s_{1}$ for Dark Urine, $s_{2}$ for Joint Pain, $s_{3}$ for Jaundice, $s_{4}$ for Abdominal Pain, $s_{5}$ for Loss of Appetite, $s_{6}$ for Fever, $s_{7}$ for Itchy Skin, and $s_{8}$ for Clay-Colored Bowel Movements. We define a relational set $R=S_{1}\times S_{2}\times S_{3}\times S_{4}$, encompassing a collection of 4-tuples, $R=\{r_{1},r_{2},r_{3},\ldots ,r_{12}\}$, each $r_{i}$, where *i* ∈ {1, 2, 3, …, 12}, uniquely representing a combination of symptoms pertinent to our diagnostic evaluation.

**Stage 1.** Following this, we construct three NHSS frameworks with the guidance of healthcare experts, specifically designed for individuals suspected Hepatitis.
  $$\begin{array}{c} \Gamma_{A}=\{\begin{array}{cc} & \{\left(r_{1},\left\{\left(\zeta_{1},0.3,0.1,0.8\right),\left(\zeta_{2},0.2,0.6,0.5\right),\left(\zeta_{3},0.3,0.7,0.4\right)\right\}\right),\\ & (r_{2},\left\{\left(\zeta_{1},0.5,0.3,0.9\right),\left(\zeta_{2},0.4,0.6,0.8\right),\left(\zeta_{3},0.1,0.5,0.7\right)\right\}),\\ & (r_{3},\left\{\left(\zeta_{1},0.3,0.4,0.7\right),\left(\zeta_{2},0.2,0.5,0.6\right),\left(\zeta_{3},0.2,0.4,0.5\right)\right\}),\\ & (r_{4},\left\{\left(\zeta_{1},0.2,0.4,0.7\right),\left(\zeta_{2},0.7,0.4,0.4\right),\left(\zeta_{3},0.4,0.2,0.8\right)\right\}),\\ & (r_{5},\left\{\left(\zeta_{1},0.3,0.6,0.9\right),\left(\zeta_{2},0.2,0.4,0.9\right),\left(\zeta_{3},0.2,0.5,0.6\right)\right\}),\\ & (r_{6},\left\{\left(\zeta_{1},0.4,0.3,0.7\right),\left(\zeta_{2},0.4,0.1,0.8\right),\left(\zeta_{3},0.2,0.3,0.5\right)\right\}),\\ & (r_{7},\left\{\left(\zeta_{1},0.3,0.6,0.5\right),\left(\zeta_{2},0.1,0.4,0.2\right),\left(\zeta_{3},0.2,0.6,0.5\right)\right\}),\\ & (r_{8},\left\{\left(\zeta_{1},0.4,0.7,0.6\right),\left(\zeta_{2},0.4,0.9,0.8\right),\left(\zeta_{3},0.1,0.4,0.7\right)\right\}),\\ & (r_{9},\left\{\left(\zeta_{1},0.4,0.6,0.7\right),\left(\zeta_{2},0.2,0.5,0.9\right),\left(\zeta_{3},0.2,0.4,0.7\right)\right\}),\\ & (r_{10},\left\{\left(\zeta_{1},0.4,0.3,0.7\right),\left(\zeta_{2},0.1,0.5,0.7\right),\left(\zeta_{3},0.2,0.5,0.7\right)\right\}),\\ & (r_{11},\left\{\left(\zeta_{1},0.1,0.2,0.4\right),\left(\zeta_{2},0.5,0.2,0.6\right),\left(\zeta_{3},0.4,0.3,0.6\right)\right\}),\\ & \left(r_{12},\left\{\left(\zeta_{1},0.6,0.9,0.1\right),\left(\zeta_{2},0.4,0.3,0.5\right),\left(\zeta_{3},0.5,0.7,0.9\right)\right\})\right\} \end{array}\}. \end{array}$$ (18)
  $$\begin{array}{c} \Lambda_{B}=\{\begin{array}{cc} & \{\left(r_{1},\left\{\left(\zeta_{1},0.4,0.5,0.9\right),\left(\zeta_{2},0.1,0.4,0.6\right),\left(\zeta_{3},0.6,0.1,0.7\right)\right\}\right),\\ & (r_{2},\left\{\left(\zeta_{1},0.7,0.2,0.8\right),\left(\zeta_{2},0.5,0.8,0.9\right),\left(\zeta_{3},0.6,0.2,0.6\right)\right\}),\\ & (r_{3},\left\{\left(\zeta_{1},0.4,0.1,0.8\right),\left(\zeta_{2},0.5,0.2,0.8\right),\left(\zeta_{3},0.6,0.6,0.1\right)\right\}),\\ & (r_{4},\left\{\left(\zeta_{1},0.7,0.4,0.9\right),\left(\zeta_{2},0.2,0.5,0.8\right),\left(\zeta_{3},0.7,0.1,0.9\right)\right\}),\\ & (r_{5},\left\{\left(\zeta_{1},0.5,0.8,0.2\right),\left(\zeta_{2},0.6,0.9,0.1\right),\left(\zeta_{3},0.7,0.4,0.8\right)\right\}),\\ & (r_{6},\left\{\left(\zeta_{1},0.6,0.1,0.2\right),\left(\zeta_{2},0.6,0.6,0.9\right),\left(\zeta_{3},0.1,0.6,0.7\right)\right\}),\\ & (r_{7},\left\{\left(\zeta_{1},0.3,0.7,0.8\right),\left(\zeta_{2},0.3,0.8,0.2\right),\left(\zeta_{3},0.4,0.7,0.7\right)\right\}),\\ & (r_{8},\left\{\left(\zeta_{1},0.6,0.2,0.7\right),\left(\zeta_{2},0.4,0.7,0.2\right),\left(\zeta_{3},0.6,0.7,0.9\right)\right\}),\\ & (r_{9},\left\{\left(\zeta_{1},0.5,0.1,0.8\right),\left(\zeta_{2},0.1,0.2,0.7\right),\left(\zeta_{3},0.2,0.4,0.7\right)\right\}),\\ & (r_{10},\left\{\left(\zeta_{1},0.4,0.3,0.7\right),\left(\zeta_{2},0.1,0.5,0.7\right),\left(\zeta_{3},0.2,0.5,0.7\right)\right\}),\\ & (r_{11},\left\{\left(\zeta_{1},0.2,0.1,0.2\right),\left(\zeta_{2},0.6,0.4,0.2\right),\left(\zeta_{3},0.5,0.1,0.2\right)\right\}),\\ & \left(r_{12},\left\{\left(\zeta_{1},0.4,0.2,0.6\right),\left(\zeta_{2},0.2,0.5,0.4\right),\left(\zeta_{3},0.1,0.2,0.5\right)\right\})\right\} \end{array}\}. \end{array}$$ (19)
  $$\begin{array}{c} {ϒ}_{C}=\{\begin{array}{cc} & \{\left(r_{1},\left\{\left(\zeta_{1},0.3,0.1,0.8\right),\left(\zeta_{2},0.2,0.6,0.5\right),\left(\zeta_{3},0.3,0.7,0.4\right)\right\}\right),\\ & (r_{2},\left\{\left(\zeta_{1},0.5,0.3,0.9\right),\left(\zeta_{2},0.4,0.6,0.8\right),\left(\zeta_{3},0.1,0.5,0.7\right)\right\}),\\ & (r_{3},\left\{\left(\zeta_{1},0.3,0.4,0.7\right),\left(\zeta_{2},0.2,0.5,0.6\right),\left(\zeta_{3},0.2,0.4,0.5\right)\right\}),\\ & (r_{4},\left\{\left(\zeta_{1},0.2,0.4,0.7\right),\left(\zeta_{2},0.7,0.4,0.4\right),\left(\zeta_{3},0.4,0.2,0.8\right)\right\}),\\ & (r_{5},\left\{\left(\zeta_{1},0.3,0.6,0.9\right),\left(\zeta_{2},0.2,0.4,0.9\right),\left(\zeta_{3},0.2,0.5,0.6\right)\right\}),\\ & (r_{6},\left\{\left(\zeta_{1},0.4,0.3,0.7\right),\left(\zeta_{2},0.4,0.1,0.8\right),\left(\zeta_{3},0.2,0.3,0.5\right)\right\}),\\ & (r_{7},\left\{\left(\zeta_{1},0.3,0.6,0.5\right),\left(\zeta_{2},0.1,0.4,0.2\right),\left(\zeta_{3},0.2,0.6,0.5\right)\right\}),\\ & (r_{8},\left\{\left(\zeta_{1},0.4,0.7,0.6\right),\left(\zeta_{2},0.4,0.9,0.8\right),\left(\zeta_{3},0.1,0.4,0.7\right)\right\}),\\ & (r_{9},\left\{\left(\zeta_{1},0.4,0.6,0.7\right),\left(\zeta_{2},0.2,0.5,0.9\right),\left(\zeta_{3},0.2,0.4,0.7\right)\right\}),\\ & (r_{10},\left\{\left(\zeta_{1},0.4,0.3,0.7\right),\left(\zeta_{2},0.1,0.5,0.7\right),\left(\zeta_{3},0.2,0.5,0.7\right)\right\}),\\ & (r_{11},\left\{\left(\zeta_{1},0.1,0.2,0.4\right),\left(\zeta_{2},0.5,0.2,0.6\right),\left(\zeta_{3},0.4,0.3,0.6\right)\right\}),\\ & \left(r_{12},\left\{\left(\zeta_{1},0.7,0.2,0.1\right),\left(\zeta_{2},0.7,0.1,0.4\right),\left(\zeta_{3},0.6,0.2,0.3\right)\right\})\right\} \end{array}\}. \end{array}$$ (20)
**Stage 2.** The system is described as follows, with another NHSS already included in the system as a reference point
  $$\begin{array}{c} \Psi_{D}=\{\begin{array}{cc} & \{\left(r_{1},\left\{\left(\zeta_{1},0.1,0.3,0.4\right),\left(\zeta_{2},0.2,0.4,0.9\right),\left(\zeta_{3},0.1,0.6,0.5\right)\right\}\right),\\ & (r_{2},\left\{\left(\zeta_{1},0.5,0.3,0.9\right),\left(\zeta_{2},0.4,0.6,0.8\right),\left(\zeta_{3},0.1,0.5,0.7\right)\right\}),\\ & (r_{3},\left\{\left(\zeta_{1},0.3,0.2,0.7\right),\left(\zeta_{2},0.2,0.7,0.6\right),\left(\zeta_{3},0.2,0.4,0.5\right)\right\}),\\ & (r_{4},\left\{\left(\zeta_{1},0.2,0.4,0.7\right),\left(\zeta_{2},0.7,0.4,0.8\right),\left(\zeta_{3},0.4,0.2,0.8\right)\right\}),\\ & (r_{5},\left\{\left(\zeta_{1},0.4,0.2,0.8\right),\left(\zeta_{2},0.1,0.6,0.7\right),\left(\zeta_{3},0.2,0.5,0.6\right)\right\}),\\ & (r_{6},\left\{\left(\zeta_{1},0.5,0.2,0.6\right),\left(\zeta_{2},0.4,0.4,0.2\right),\left(\zeta_{3},0.2,0.3,0.5\right)\right\}),\\ & (r_{7},\left\{\left(\zeta_{1},0.3,0.9,0.3\right),\left(\zeta_{2},0.2,0.6,0.7\right),\left(\zeta_{3},0.2,0.6,0.5\right)\right\}),\\ & (r_{8},\left\{\left(\zeta_{1},0.4,0.3,0.8\right),\left(\zeta_{2},0.4,0.9,0.8\right),\left(\zeta_{3},0.1,0.4,0.7\right)\right\}),\\ & (r_{9},\left\{\left(\zeta_{1},0.4,0.6,0.7\right),\left(\zeta_{2},0.2,0.5,0.2\right),\left(\zeta_{3},0.2,0.4,0.7\right)\right\}),\\ & (r_{10},\left\{\left(\zeta_{1},0.4,0.3,0.7\right),\left(\zeta_{2},0.1,0.5,0.6\right),\left(\zeta_{3},0.2,0.5,0.7\right)\right\}),\\ & (r_{11},\left\{\left(\zeta_{1},0.4,0.1,0.4\right),\left(\zeta_{2},0.4,0.2,0.6\right),\left(\zeta_{3},0.4,0.3,0.6\right)\right\}),\\ & \left(r_{12},\left\{\left(\zeta_{1},0.5,0.7,0.3\right),\left(\zeta_{2},0.8,0.2,0.5\right),\left(\zeta_{3},0.6,0.4,0.8\right)\right\})\right\} \end{array}\}. \end{array}$$ (21)
**Stage 3-4** We assess the Hamming distances using the NHSS metric and subsequently implement the SIM as outlined below:
  - Compute the SIM of $\Gamma_{A}$ and $\Psi_{D}$:
    $$\begin{array}{c} S_{NHSS}^{\prime }\left(\Gamma_{A},\Psi_{D}\right)=\frac{1}{1+d_{NHSS}^{s}\left(\Gamma_{A},\Psi_{D}\right)}≅0.51>\frac{1}{2} \end{array}$$
  - Compute the SIM of $\Lambda_{B}$ and $\Psi_{D}$:
    $$\begin{array}{c} S_{NHSS}^{\prime }\left(\Lambda_{B},\Psi_{D}\right)=\frac{1}{1+d_{NHSS}^{s}\left(\Lambda_{B},\Psi_{D}\right)}≅0.34<\frac{1}{2} \end{array}$$
  - Compute the SIM of ${ϒ}_{C}$ and $\Psi_{D}$:
    $$\begin{array}{c} S_{NHSS}^{\prime }\left({ϒ}_{C},\Psi_{D}\right)=\frac{1}{1+d_{NHSS}^{s}\left({ϒ}_{C},\Psi_{D}\right)}≅0.64>\frac{1}{2} \end{array}$$
**Stage 5.** From this information, it is likely that the individuals *ζ*_1_ and *ζ*_3_ are affected by Hepatitis.

### 3.3 Methodology III

#### 3.3.1 Numerical example

Our aim is to ascertain the most impactful infectious disease in North America, focusing on four primary diseases; We obtain medical images from individuals suspected of having infectious diseases and use the technique outlined in Step 2 to convert these images into the NHSS format. We seek to determine their respective degrees of impact, ranging from the most to the least disruptive. The methodology initiates with the acquisition of chest images from affected individuals, which are subsequently transformed into NHSS format, adhering to the methodology delineated in Eqs 16 and 22. Our goal, with the guidance of experts, is to discern which of these diseases exerts the most significant influence on the population, as illustrated in Table 1. The initial step involves processing the NHSS data displayed in Table 1, converting it to Fuzzy Hypersoft Set (HSS) data as explicated in Table 2. Upon completing this conversion, we utilize the Technique for Order Preference by Similarity to Ideal Solution (TOPSIS) method to identify the disease that represents the foremost threat.

**Table 1:** All experts opinions collectively (NHSS values).

Types of Diseases	$H_{1}$	$H_{2}$	$H_{3}$	$H_{4}$
Syphilis	(0.6, 0.1, 0.4)	(0.5, 0.1, 0.7)	(0.1, 0.1, 0.2)	(0.4, 0.6, 0.1)
Lyme Disease	(0.6, 0.2, 0.8)	(0.2, 0.6, 0.4)	(0.5, 0.7, 0.4)	(0.2, 0.1, 0.5)
Pertussis	(0.4, 0.6, 0.3)	(0.1, 0.2, 0.6)	(0.7, 0.2, 0.4)	(0.1, 0.5, 0.3)
Norovirus	(0.2, 0.7, 0.9)	(0.5, 0.1, 0.7)	(0.2, 0.1, 0.6)	(0.7, 0.1, 0.6)

**Table 2:** All experts opinions collectively (FHS values).

Types of Diseases	$H_{1}$	$H_{2}$	$H_{3}$	$H_{4}$
Syphilis	0.41	0.6	0.47	0.34
Lyme Disease	0.10	0.18	0.04	0.28
Pertussis	0.56	0.30	0.12	0.38
Norovirus	0.74	0.34	0.78	0.23

**Algorithm 2** Diagnostic Algorithm Phase I

1: **Input:** Registered input images *I*

2: **Output:** Fuzzy Hypersoft Set transformation of the input images

3: **Step 1: Fuzzification**

4: First, the initial input image undergoes fuzzification through the application of Eq 22.

$$\begin{array}{c} \mu_{I_{1}}\left(I_{gh_{1}}\right)=\frac{I_{gh_{1}}-I_{min}}{I_{max}-I_{min}}, \end{array}$$ (22)

where $I_{gh_{1}}$ is the gray pixel of the first input image. *I*_*max*_ and *I*_*min*_ represent the highest and least gray level pixel values of the first input image, respectively.

5: **Step 2: Neutrosophic Hypersoft Set (NHSS) Development**

6: To develop an NHSS based on the provided images, follow these sub-steps:

- **Membership, Non-membership, and Indeterminacy Degree Construction**:
  The degrees are defined by:
  $$\begin{array}{c} \left(\mu_{I_{1}}^{NIFS},\nu_{I_{1}}^{NIFS},\gamma_{I_{1}}^{NIFS}\right):\left(I_{gh_{1}};\beta_{opt1}\right)\mapsto \left[0,1\right], \end{array}$$ (23)
  $$\begin{array}{c} 0\leq \mu_{I_{1}}^{NIFS}+\nu_{I_{1}}^{NIFS}+\gamma_{I_{1}}^{NIFS}\leq 3 \end{array}$$ (24)
  $$\begin{array}{c} \left(I_{gh_{1}};\beta_{opt1}\right)=\frac{\left(1+\beta_{opt1}\left(1+e^{-\beta_{opt1}}\right)\right)\mu_{I_{1}}\left(I_{gh1}\right)}{\left(1+\beta_{opt1}\left(1+e^{-\beta_{opt1}}\right)\right)\mu_{I_{1}}\left(I_{gh1}\right)} \end{array}$$ (25)
- **System Representation:**
  The system can be represented as:
  $$\begin{array}{c} I_{IF_{1}}=\left\{I_{gh_{1}},\mu_{I_{1}}^{NIFS}\left(I_{gh_{1}};\beta_{opt1}\right),\nu_{I_{1}}^{NIFS}\left(I_{gh_{1}};\beta_{opt1}\right),\gamma_{I_{1}}^{NIFS}\left(I_{gh_{1}};\beta_{opt1}\right)\right\} \end{array}$$ (26)
- **Optimization Parameter:**
  The optimal parameter *β*_*opt*1_ is determined by maximizing the Pythagorean Fuzzy Entropy (PFE):
  $$\begin{array}{c} \beta_{opt1}=\underset{\beta }{\text{max}}\left(PFE\left(F;\beta \right)\right) \end{array}$$ (27)
  where PFE is calculated as:
  $$\begin{array}{c} PFE\left(F;\beta \right)=\frac{1}{n}\sum_{i=1}^{n}\pi_{F}\left(p_{i}\right)e^{\pi_{F}\left(p_{i}\right)} \end{array}$$ (28)

7: **Step 3: Transformation to Fuzzy Hypersoft Set**

8: Transform NHSS into a Fuzzy Hypersoft Set by applying the following formula:

$$\begin{array}{c} \sigma_{fuzzy}\left(x\right)=\frac{1}{2}\left[T-2I-F\right]\;\left[33\right] \end{array}$$ (29)

**Algorithm 3** Diagnostic Algorithm Phase II

1: **Input:** Decision matrix *x*, weights *w*_*i*_ for criteria *i* = 1, 2, …, *m*

2: **Output:** Ranked alternatives based on preference values *V*_*i*_ for *i* = 1, 2, …, *m*

3: **Objective:** To create a decision average matrix for each alternative based on the collective perspective of professionals in the NHSS, utilizing the standardized precipitation fuzzy conceptual framework. This decision matrix is widely recognized in the field.

4: **Step 1: Normalize the Decision Matrix**

5:  Normalize the decision matrix *x* to create a normalized decision matrix *r*_*ij*_ using Eq 30, where *i* = 1, 2, …, *m* and *j* = 1, 2, …, *n*.

$$\begin{array}{c} r_{ij}=\frac{x_{ij}}{\sqrt{\sum_{i=1}^{m}x_{ij}^{2}}} \end{array}$$ (30)

6: **Step 2: Compute the Weighted Normalized Decision Matrix**

7:  Based on the weighted normalized rating *y*_*ij*_, compute the weighted normalized decision matrix as shown in Eq 31.

$$\begin{array}{c} y_{ij}=r_{ij}w_{i} \end{array}$$ (31)

8: **Step 3: Determine Positive and Negative Ideal Solutions**

9:  Formulate matrices for the positive ideal solution (*A*^+^) and the negative ideal solution (*A*^−^) using Eqs 32 and 33.

$$\begin{array}{c} A^{+}=\left(y_{1}^{+},y_{2}^{+},y_{3}^{+},\ldots ,y_{n}^{+}\right) \end{array}$$ (32)

$$\begin{array}{c} A^{-}=\left(y_{1}^{-},y_{2}^{-},y_{3}^{-},\ldots ,y_{n}^{-}\right) \end{array}$$ (33)

10: **Step 4: Calculate the Distance to Ideal Solutions**

11:  Compute the distance of each alternative *A*_*i*_ from the positive ideal solution *A*^+^ using Eq 34, where *i* = 1, 2, …, *m*.

$$\begin{array}{c} D_{i}^{+}=\sqrt{\sum_{j=1}^{n}{\left(y_{ij}-y_{j}^{+}\right)}^{2}} \end{array}$$ (34)

12:  Similarly, calculate the distance from the negative ideal solution *A*^−^ using Eq 35.

$$\begin{array}{c} D_{i}^{-}=\sqrt{\sum_{j=1}^{n}{\left(y_{ij}-y_{j}^{-}\right)}^{2}} \end{array}$$ (35)

13: **Step 5: Calculate the Preference Value for Each Alternative**

14:  Determine the value of preference *V*_*i*_ for each alternative using Eq 36, where *i* = 1, 2, …, *m*.

$$\begin{array}{c} V_{i}=\frac{D_{i}^{-}}{D_{i}^{+}+D_{i}^{-}} \end{array}$$ (36)

15: **Step 6: Rank the Alternatives**

16:  Arrange the choices based on the preference values *V*_*i*_ and select the most suitable one.

1. **Step 1:** Consider the ensemble X constituted by the elements {*ζ*_1_, *ζ*_2_, *ζ*_3_, *ζ*_4_}, defined as follows: *ζ*_1_ represents the Syphilis, *ζ*_2_ is indicative of Lyme Disease, *ζ*_3_ corresponds to Pertussis, and *ζ*_4_ encapsulates Norovirus. The collection of sub-parametric triplets associated with the symptomatic parameter values for each disease are denoted by $H_{1}$, $H_{2}$, $H_{3}$, and $H_{4}$, as elaborated within Table 2. Following this, normalization of Table 3 is accomplished in accordance with Eq (30).
2. **Step 2:** Utilizing Eq (31), a weighted decision matrix is developed for the alternatives, as demonstrated in Table 3, with the corresponding weighted decision matrix presented in Table 4.
3. **Step 3:** The positive ideal solution and the negative ideal solution are determined by applying Eqs (32) and (33), respectively, with the results displayed in Tables 5 and 6.
4. **Step 4:** Calculate the distance of each option from the positive and negative ideal solutions utilizing Eqs (34) and (35), as indicated in Tables 7 and 8.
5. **Step 5:** Derive the preference value for each option using Eq (36), as detailed in Table 9.
6. **Step 6:** Rank the options to identify the most favorable one, as presented in Table 10.

**Table 3:** Normalized matrix.

Types of Diseases	$H_{1}$	$H_{2}$	$H_{3}$	$H_{4}$
Syphilis	0.52637	0.62732	0.37482	0.38273
Lyme Disease	0.29332	0.72392	0.62381	0.82739
Pertussis	0.72812	0.52732	0.72837	0.37293
Norovirus	0.72839	0.q	0.82336	0.34229

**Table 4:** Weighted normalized matrix.

Types of Diseases	$H_{1}$	$H_{2}$	$H_{3}$	$H_{4}$
Syphilis	0.03107	0.28355	0.10978	0.1141
Lyme Disease	0.01102	0.09641	0.01193	0.08802
Pertussis	0.05713	0.1758	0.022625	0.12062
Norovirus	0.07517	0.19849	0.16467	0.06846

**Table 5:** Positive ideal solution.

Types of Diseases	Ideal Positive Values
$C_{1}$	0.01102
$C_{2}$	0.09641
$C_{3}$	0.01193
$C_{4}$	0.06846

**Table 6:** Negative ideal solution.

Types of Diseases	Ideal Negative Values
$C_{1}$	0.07517
$C_{2}$	0.28355
$C_{3}$	0.16467
$C_{4}$	0.12062

**Table 7:** Euclidean distance from ideal positive.

Types of Diseases	Separation Values
Syphilis	0.21698
Lyme Disease	0.01956
Pertussis	0.10656
Norovirus	0.19458

**Table 8:** Euclidean distance from ideal negative.

Types of Diseases	Separation Values
Syphilis	0.07071
Lyme Disease	0.25205
Pertussis	0.17634
Norovirus	0.09978

**Table 9:** Preference values.

Types of Diseases	Values
Syphilis	0.561
Lyme Disease	0.872
Pertussis	0.563
Norovirus	0.328

**Table 10:** Final ranking matrix.

Types of Diseases	$H_{1}$	$H_{2}$	$H_{3}$	$H_{4}$	Rank
Syphilis	0.29	0.51	0.48	0.33	3
Lyme Disease	0.9	0.18	0.06	0.29	1
Pertussis	0.61	0.32	0.12	0.39	2
Norovirus	0.78	0.32	0.81	0.19	4

#### 3.2.2 Proposed method’s algorithm

Let’s consider a group, *U*, representing individuals who could potentially be infected with the MERS. Suppose $R=\{s_{1},s_{2},\ldots ,s_{k}\}\subset U$ represents a subgroup comprising individuals currently being scrutinized. Let $Q=\{\beta_{1},\beta_{2},\ldots ,\beta_{n}\},n\geq 1$, symbolize a collection of symptoms or attributes, and let *B* = {*β*_1_, *β*_2_, …, *β*_*m*_}, *m* ≤ *n*, where B⊂Q, represent a subset of these symptoms that are currently under analysis. The Cartesian product *C* = *A*_1_ × *A*_2_ × ⋯ × *A*_*n*_ is formed, where *A*_*i*_ for *i* = 1, 2, …, *n* pertains to the set of possible outcomes for attribute *β*_*i*_. It’s crucial that these sets are exclusive of one another, ensuring *A*_*i*_∩*A*_*j*_ = ∅ for any *i*, *j* ∈ 1, 2, …, *n*. For every individual *s* in R, a relationship *d*(*s*, *δ*) is established, showcasing the degree of connection of *s* to each attribute value *δ*. This relationship can be articulated in terms of fuzzy, Pythagorean fuzzy, or neutrosophic logic. The aim for medical practitioners is to leverage this model to gauge the probability of MERS infection among the scrutinized individuals and ascertain the infection’s intensity, thereby facilitating an informed decision-making process regarding each case. The subsequent sections will elaborate on the AI-based methodology employed in developing this algorithm for such assessments.

**Algorithm 4** Diagnostic Algorithm

**Step 1: Assign Fuzzy Membership** In the Plithogenic Hypersoft Set (PHSS) $R_{1}$, the specialist assigns a fuzzy membership degree to each suspect in relation to each symptom. This degree represents the likelihood or degree to which a suspect exhibits a particular symptom.

**Step 2: Organize Disease Symptoms** Organize the disease symptoms under investigation into a set based on their established values. Group these symptoms according to sub-symptoms and categorize each by severity in another PHSS, denoted as $R_{2}$.

**Step 3: Evaluate Distance Measures** Utilize the newly proposed distance measures, namely the Plithogenic Hamming Distance (PHD) measure and the Plithogenic Euclidean Distance (PED) measure, to evaluate the distance between PHSS $R_{1}$ and $R_{2}$.

$$\begin{array}{c} d_{H}^{R}(R_{1},R_{2})=\frac{1}{m}\sum_{i=1}^{m}\sum_{j=1}^{l}|d_{R_{1}}^{i}(\delta_{j})-d_{R_{2}}^{i}(\delta_{j})|\times \text{max}(c_{F}^{i}(\delta_{j},\delta_{d})) \end{array}$$ (37)

$$\begin{array}{c} d_{E}^{R}(R_{1},R_{2})={\left[\frac{1}{m}\sum_{i=1}^{m}\sum_{j=1}^{l}{\left(d_{R_{1}}^{i}(\delta_{j})-d_{R_{2}}^{i}(\delta_{j})\right)}^{2}\times \text{max}(c_{F}^{i}(\delta_{j},\delta_{d}))\right]}^{\frac{1}{2}} \end{array}$$ (38)

**Step 4: Calculate Plithogenic Similarity Measure (PSM)** Calculate the PSM by determining the Plithogenic Distance Measure (PDM) between values assigned by experts and the corresponding standard values to evaluate their resemblance.

$$\begin{array}{c} M^{R}=M^{R}(R_{1},R_{2})=\frac{1}{1+d^{R}(R_{1},R_{2})} \end{array}$$ (39)

**Step 5: Diagnose Infection** If $M^{R}<0.5$, the suspect is not infected. If $M^{R}\geq 0.5$, the individual may be infected. Further actions depend on the level of infection severity.

- If $0.5\leq M^{R}<0.7$, isolate and quarantine the suspect at home.
- If $0.7\leq M^{R}<0.9$, isolate the suspect in a designated quarantine facility.
- If $0.9\leq M^{R}\leq 1$, dispatch the suspect to the hospital for appropriate medical care.

  **Remark:** Thresholds mentioned were based on hypothetical data for creating a prototype and conducting diagnostic tests. Adjustments can be made with real data and medical professional guidance.

### 3.4 Implementation of the proposed method

Consider a defined universe *U*, within which lies a subset $R=\{s_{1},s_{2},...,s_{5}\}$ comprising individuals suspected of being infected with MERS. Their assessment is based on monitoring various symptoms or attributes, listed as follows:

*β*_1_ Fever
*β*_2_ Dry cough
*β*_3_ Tiredness
*β*_4_ Breathing difficulty or breath shortness
*β*_5_ Chest pain or pressure
*β*_6_ Loss of speech or movement
*β*_7_ Aches and pains
*β*_8_ Throat discomfort
*β*_9_ Diarrhea
*β*_10_ Conjunctivitis
*β*_11_ Headache
*β*_12_ Diminished ability to taste or smell
*β*_13_ Skin rash or changes in finger/toe color

To gauge symptom intensity, each *β*_*i*_ is classified into three severity tiers—low (*L*) for mild symptoms, medium (*M*) for moderate symptoms, and high (*H*) for severe symptoms. This aids in diagnosis by assigning specific severity levels as follows:

*δ*(*i*, 1) = Low,
*δ*(*i*, 2) = Medium,
*δ*(*i*, 3) = High,

for 1 ≤ *i* ≤ 13. The observed subset of symptoms in suspects from a particular area includes *β*_1_, *β*_2_, *β*_3_, *β*_4_, *β*_5_, *β*_6_, *β*_9_, *β*_11_. For each suspect, hypothetical fuzzy membership degrees are assigned for these symptoms, under specific constraints defined by experts, as shown in Table 11. Each selected symptom has a predominant attribute value *δ*_*d*_ and a fuzzy contradiction degree *c*_*F*_ reflecting the discrepancy between actual and predominant values, detailed in Table 12. A PHSS, denoted as $R_{1}$, is developed to systematically represent this information, incorporating expert medical opinions (see Table 13). The expert-assigned values for the selected symptoms are documented accordingly (see Table 14). To assess the discrepancy between specialist-recorded values and established benchmarks. Following this, the investigation evaluates plithogenic similarity, leveraging plithogenic distance, to gauge the extent of infection in the observed subjects. Details on how plithogenic similarity is calculated are provided in Table 15. Drawing from the insights in Table 15, the study efficiently extracts values for both Hamming and Euclidean similarity measures. These values inform the subsequent decisions made regarding the suspects, which are detailed in Table 16. For more detail, please see figure file.

**Table 11:** Degree of belongingness of each option with respect to each attribute value, measured in fuzzy terms.

Symptoms	Severity	Suspects				
		*s*_1_	*s*_2_	*s*_3_	*s*_4_	*s*_5_
*β*_9_	Low	0.57	0.32	0.23	0.49	0.27
	Medium	0.67	0.47	0.31	0.61	0.34
	High	0.98	0.91	0.52	0.69	0.42
*β*_2_	Low	0.49	0.49	0.51	0.31	0.19
	Medium	0.61	0.67	0.54	0.51	0.32
	High	0.77	0.70	0.67	0.51	0.62
*β*_2_	Low	0.51	0.29	0.19	0.21	0.39
	Medium	0.67	0.33	0.41	0.29	0.54
	High	0.79	0.47	0.47	0.55	0.70
*β*_3_	Low	0.51	0.39	0.21	0.47	0.21
	Medium	0.61	0.45	0.67	0.56	0.28
	High	0.63	0.48	0.70	0.58	0.41
*β*_4_	Low	0.54	0.41	0.20	0.51	0.56
	Medium	0.57	0.42	0.19	0.67	0.58
	High	0.73	0.45	0.32	0.76	0.59
*β*_5_	Low	0.65	0.45	0.41	0.60	0.23
	Medium	0.67	0.49	0.42	0.82	0.34
	High	0.78	0.78	0.49	0.73	0.56
*β*_6_	Low	0.45	0.13	0.15	0.21	0.23
	Medium	0.48	0.23	0.23	0.38	0.27
	High	0.49	0.29	0.34	0.39	0.29
*β*_7_	Low	0.21	0.29	0.19	0.19	0.22
	Medium	0.34	0.29	0.37	0.39	0.31
	High	0.29	0.49	0.61	0.29	0.39
*β*_8_	Low	0.54	0.67	0.65	0.43	0.46
	Medium	0.62	0.65	0.80	0.56	0.51
	High	0.61	0.87	0.18	0.51	0.58
*β*_9_	Low	0.23	0.24	0.21	0.26	0.26
	Medium	0.31	0.21	0.24	0.43	0.34
	High	0.45	0.43	0.45	0.45	0.47
*β*_10_	Low	0.45	0.65	0.24	0.21	0.27
	Medium	0.47	0.67	0.28	0.44	0.29
	High	0.67	0.70	0.39	0.67	0.47
*β*_11_	Low	0.19	0.23	0.28	0.45	0.21
	Medium	0.26	0.45	0.29	0.47	0.27
	High	0.43	0.28	0.41	0.51	0.38
*β*_12_	Low	0.41	0.43	0.42	0.26	0.54
	Medium	0.45	0.51	0.45	0.31	0.57
	High	0.51	0.63	0.51	0.34	0.62

**Table 12:** Degrees of contradiction correlate with the dominant value.

Symptoms	Dominant	*L*	*M*	*H*
*β*_1_	High	0.80	0.69	0.00
*β*_2_	High	0.97	0.54	0.00
*β*_3_	Medium	0.32	0.00	0.54
*β*_4_	High	0.97	0.82	0.00
*β*_5_	High	0.96	0.76	0.00
*β*_6_	Medium	0.43	0.00	0.48
*β*_9_	Low	0.00	0.39	0.87
*β*_11_	Low	0.00	0.42	0.87

**Table 13:** Degree of affiliation assigned to the suspects by the specialist using fuzzy logic.

Symptoms	Severity	Suspects				
		*s*_1_	*s*_2_	*s*_3_	*s*_4_	*s*_5_
*β*_1_	Low	0.57	0.32	0.23	0.49	0.27
	Medium	0.67	0.47	0.31	0.61	0.34
	High	0.98	0.91	0.52	0.69	0.42
*β*_2_	Low	0.49	0.49	0.51	0.31	0.19
	Medium	0.61	0.67	0.54	0.51	0.32
	High	0.77	0.70	0.67	0.51	0.62
*β*_3_	Low	0.51	0.29	0.19	0.21	0.39
	Medium	0.67	0.33	0.41	0.29	0.54
	High	0.79	0.47	0.47	0.55	0.70
*β*_4_	Low	0.51	0.39	0.21	0.47	0.21
	Medium	0.61	0.45	0.67	0.56	0.28
	High	0.63	0.48	0.70	0.58	0.41
*β*_5_	Low	0.54	0.41	0.20	0.51	0.56
	Medium	0.57	0.42	0.19	0.67	0.58
	High	0.73	0.45	0.32	0.76	0.59
*β*_6_	Low	0.65	0.45	0.41	0.60	0.23
	Medium	0.67	0.49	0.42	0.82	0.34
	High	0.78	0.78	0.49	0.73	0.56
*β*_9_	Low	0.54	0.67	0.65	0.43	0.46
	Medium	0.62	0.65	0.80	0.56	0.51
	High	0.61	0.87	0.18	0.51	0.58
*β*_11_	Low	0.45	0.65	0.24	0.21	0.27
	Medium	0.47	0.67	0.28	0.44	0.29
	High	0.67	0.70	0.39	0.67	0.47

**Table 14:** Established criteria for selected symptoms.

Attributes	Low	Medium	High
*β*_1_	0.62	0.76	0.99
*β*_2_	0.56	0.72	0.82
*β*_3_	0.65	0.74	0.78
*β*_4_	0.65	0.79	0.87
*β*_5_	0.59	0.78	0.88
*β*_6_	0.65	0.92	98
*β*_9_	0.41	0.53	0.70
*β*_11_	0.38	0.58	0.69

**Table 15:** Similarity measures.

Suspects	$M_{H}^{R}(R_{1},R_{2})$	$M_{E}^{R}(R_{1},R_{2})$
*s*_1_	0.52	0.58
*s*_2_	0.93	0.95
*s*_3_	0.27	0.29
*s*_4_	0.72	0.82
*s*_5_	0.74	0.85

**Table 16:** Suspects decision.

Decision	Suspects
Safe zone	*s*_3_
Home Isolation	*s*_1_
Quarantine center	*s*_4_, *s*_5_
Hospital treatment	*s*_2_

### 3.5 Limitation

1. Dependence on high-quality medical images, which may not be available in all settings.
2. Potential biases in medical image data that could affect the accuracy of diagnoses.
3. Variability in healthcare infrastructure across different countries could impact the applicability of the proposed methodologies.

## 4 Conclusion

This research addresses the critical challenge of achieving precise disease diagnosis in medicine, a major obstacle to effective patient care and treatment. It investigates various factors affecting diagnostic accuracy to improve diagnostic methods, considering their impact on patient well-being and healthcare costs. The study aims to influence clinical practices, healthcare administration, and policy formulation by highlighting the importance of accurate and cost-efficient diagnostic processes. Central to this research is the development of a model to identify optimal diagnostic strategies, emphasizing collaborative research, particularly in resource-constrained environments, and demonstrating how technological advancements can enhance diagnostic accuracy. It advocates for the seamless integration of these innovations into standard clinical procedures. The paper introduces three AI-driven diagnostic models that represent a novel and systematic approach to medical diagnosis, combining advanced computational methods with Multi-Criteria Decision Making (MCDM) frameworks. The first model applies Neutrosophic Hypersoft Set (NHSS) measures to medical images, converting them to NHSS format for diagnosis. The second model uses the TOPSIS method as a robust diagnostic tool. The third model explores plithogenic distance and similarity measures within the plithogenic hypersoft sets framework, particularly for suspected SARS cases. This involves developing mathematical models to evaluate MERS suspects using an MCDM approach, integrating plithogenic hypersoft sets into a fuzzy context, and creating a unique algorithm based on these metrics. This algorithm aims to detect and assess infection severity, guiding decisions on isolation, quarantine, or hospitalization. By constructing a mathematical model capable of identifying potential infectious diseases from medical images using an MCDM approach, this research leverages AI to refine diagnostic techniques. Future directions will focus on integrating AI-based diagnostic tools with real-time data from diverse global health systems to enhance early detection and personalized treatment. Additionally, developing universally accessible platforms for these AI tools can democratize advanced healthcare solutions. Ongoing refinement of machine learning models with extensive and varied datasets will ensure their robustness and adaptability in managing infectious diseases globally.
